# Rationally Designed Dendritic Silica Nanoparticles for Oral Delivery of Exenatide

**DOI:** 10.3390/pharmaceutics11080418

**Published:** 2019-08-19

**Authors:** Muhammad Mustafa Abeer, Anand Kumar Meka, Naisarg Pujara, Tushar Kumeria, Ekaterina Strounina, Rute Nunes, Ana Costa, Bruno Sarmento, Sumaira Z. Hasnain, Benjamin P. Ross, Amirali Popat

**Affiliations:** 1 School of Pharmacy, The University of Queensland, Brisbane QLD 4072, Australia; 2 Mater Research Institute—The University of Queensland, Translational Research Institute, Woolloongabba QLD 4102, Australia; 3 Center for Advanced Imaging, The University of Queensland, Brisbane QLD 4072, Australia; 4 Instituto de Investigação e Inovação em Saúde (I3S), University of Porto, Rua Alfredo Allen, 208, 4200-135 Porto, Portugal; 5 Instituto de Engenharia Biomédica (INEB), University of Porto, Rua Alfredo Allen, 208, 4200-135 Porto, Portugal; 6 CESPU, Instituto de Investigação e Formação Avançada em Ciências e Tecnologias da Saúde, Rua Central de Gandra, 1317, 4585-116 Gandra, Portugal; 7 Australian Infectious Disease Research Centre—The University of Queensland Building 76 Room 155 Cooper Road, St. Lucia QLD 4067, Australia

**Keywords:** large pore silica nanoparticles, exenatide, oral delivery, anti-diabetic peptides

## Abstract

Type 2 diabetes makes up approximately 85% of all diabetic cases and it is linked to approximately one-third of all hospitalisations. Newer therapies with long-acting biologics such as glucagon-like peptide-1 (GLP-1) analogues have been promising in managing the disease, but they cannot reverse the pathology of the disease. Additionally, their parenteral administration is often associated with high healthcare costs, risk of infections, and poor patient adherence associated with phobia of needles. Oral delivery of these compounds would significantly improve patient compliance; however, poor enzymatic stability and low permeability across the gastrointestinal tract makes this task challenging. In the present work, large pore dendritic silica nanoparticles (DSNPs) with a pore size of ~10 nm were prepared, functionalized, and optimized in order to achieve high peptide loading and improve intestinal permeation of exenatide, a GLP-1 analogue. Compared to the loading capacity of the most popular, Mobil Composition of Matter No. 41 (MCM-41) with small pores, DSNPs showed significantly high loading owing to their large and dendritic pore structure. Among the tested DSNPs, pristine and phosphonate-modified DSNPs (PDSNPs) displayed remarkable loading of 40 and 35% *w*/*w*, respectively. Furthermore, particles successfully coated with positively charged chitosan reduced the burst release of exenatide at both pH 1.2 and 6.8. Compared with free exenatide, both chitosan-coated and uncoated PDSNPs enhanced exenatide transport through the Caco-2 monolayer by 1.7 fold. Interestingly, when a triple co-culture model of intestinal permeation was used, chitosan-coated PDSNPs performed better compared to both PDSNPs and free exenatide, which corroborated our hypothesis behind using chitosan to interact with mucus and improve permeation. These results indicate the emerging role of large pore silica nanoparticles as promising platforms for oral delivery of biologics such as exenatide.

## 1. Introduction

Peptide-based therapeutics are specific and potent in action, and typically have better safety and tolerability profiles relative to small organic molecules [1]. Their superior action is partly explained by their larger size as compared to small molecules, which facilitates greater interaction with target sites present in shallow or extended binding pockets [2]. However, oral delivery of peptides remains challenging due to the chemical and enzymatic hydrolysis and poor absorption, and poor drug likeness (peptide hydrophilicity and high molecular weight) [3,4]. Despite these hurdles, the pursuit to develop oral drug delivery systems for many proteins and peptides has been ongoing for many decades but without clinically approved oral formulation of peptides and proteins.

Incretins such as GLP-1 are important regulators of metabolic homeostasis which are released by intestinal *L* cells in response to glucose and other ingested nutrients [5]. GLP-1 analogues are available in the form of parenteral formulations aimed at enhancing insulin secretion and limiting the release of stored sugar in a glucose-dependent manner and, hence, reducing the risk of hypoglycemia [6]. Oral peptide-based medicines in this category are still under development and so far one oral formulation of semaglutide from Novo Nordisk has recently completed Phase III trial [7]. Therefore, numerous approaches are being investigated to overcome the challenges posed by the gastrointestinal tract (GIT) in effective oral delivery of peptides and protein therapeutics. In the majority of the approaches, nano-or/micro particulate systems have been used. For instance, the chitosan-coated porous silicon nanoparticles have been shown to control the release of GLP-1 for a period pertaining to gastric transit time and the composite of chitosan and inorganic nanoparticles have been preferred over organic particle systems like poly(lactide-*co*-glycolide) (PLGA) and solid lipid nanoparticles due to the poor loading of the organic NPs [8]. Chitosan was also used in order to improve mucus and cellular adhesion leading to increased retention time at the site of absorption. The loading capacity (LC) of the silicon-based system was reported to be 17% which was ~100 fold higher than the polymeric nanoparticles tested in the study. The difference was attributed to the process involved in loading, as the payload was retained within pores of porous silicon nanoparticles following physical adsorption while GLP-1 was entrapped within nanoparticles as the particles were being produced in double emulsion systems causing possible loss of the peptide during preparation. Recently, self-emulsifying drug delivery systems of exenatide with ~45 nm in size have been prepared by lipophilic modification of exenatide [9]. However, the system made use of significant amounts of surfactants to perform hydrophobic ion pairing and exenatide payload accounted for only 0.71% *w*/*w* [10,11].

Silica nanoparticles (SNPs) are emerging functional inorganic nanomaterials which offer an alternative to traditional carriers for oral delivery of peptides and drugs [12,13,14,15]. Modification of SNPs with appropriate functional groups improves control over the loading/release of payload for oral delivery. As an alternative strategy, porous SNPs can also be coated with a polymer as a gate-keeper for encapsulated peptide [16,17]. Non-porous and porous silica nanoparticles have been used for potential oral delivery of another antidiabetic peptide, insulin [18,19]. However, non-porous solid silica or traditional mesoporous silica nanoparticles (Mobil Composition of Matter No. 41 and 48; MCM-41, MCM-48) have no or small pore size of 2–3 nm, respectively, and offer limited space for large peptide or protein to be encapsulated. Hence, focus has shifted towards large pore silica structures (10 nm or higher) in order to achieve high loading with room for further modification [20,21,22]. Chen et al. [23] showed evidence of controlled release of exenatide using SBA-15 type silica microparticles at pH 7.4. However, this study investigated intravenous administration of exenatide-loaded microparticles. Use of such large pore structures have been very popular in the field of enzyme therapies, vaccine delivery, and imaging [24,25,26,27]. To the best of our knowledge, applicability of large pore dendritic silica nanoparticle (DSNPs) to deliver exenatide has not been studied.

We hypothesized that DSNPs will enable high loading of exenatide and potentially improve permeation of exenatide across the intestinal barrier. The current study is aimed at screening of different functionalized DSNPs in terms of physiochemical, textural, and loading characteristics and finding a candidate suitable for oral delivery of exenatide and other similar macromolecular payloads. Herein, large pore DSNPs were synthesized with various functional groups, characterized and screened for optimal loading of exenatide. Phosphonate-modified DSNPs with high loading were coated with chitosan to improve permeation of exenatide in vitro using a Caco-2 model and a triple co-culture model of intestinal permeability. Our proof of concept study demonstrates the potential of large pore silica nanoparticles as an alternative carrier for oral delivery of exenatide and calls for in vivo testing.

## 2. Materials and Methods

### 2.1. Synthesis of Dendritic Silica Nanoparticles

Pristine DSNPs were prepared by following the protocol of Xu et al. with slight modification [28]. Typically, 0.18 g (~0.16 mL) of triethanolamine (TEA) was weighed into a 200 mL glass bottle. Deionized water (36 mL) and 25 wt% of cetyltrimethylammonium chloride in water (CTAC, 24 mL) were added and the mixture was stirred for 1 h at 500 rpm and 60 °C in the oil bath. After 1 h, 52.5 mL of chlorobenzene and 7.5 mL tetraethylorthosilicate (TEOS) were pre-mixed and then added to the aqueous phase. The milky suspension was stirred for another 18 h. After 18 h, the prepared DSNPs were sedimented and supernatant was removed after running at 24,700× *g* for 10 min at room temperature (rt). The particles were re-dispersed into excess of ethanol using bath sonication (Branson 2800, 40 kHz, Branson, Danbury, CA, USA) for 5–10 min and then centrifuged again at the same speed. Washing steps were repeated for water and ethanol and separated DSNPs were dried overnight at 80 °C. After drying, particles were calcined at 550 °C for 6 h in a furnace. After the process, around 0.75 g DSNPs were obtained and stored for future use in air-tight containers. To understand the role of surface chemistry on loading and release of exenatide, four different surface functional groups were imparted onto MSNs.

### 2.2. Synthesis of Phosphonate Modified DSNPs (PDSNPs)

Pristine DSNPs (100 mg) were dispersed in 13 mL of Milli-Q water using a bath sonicator for 3 min. Phosphonate silane 3-(trihydroxysilyl)propyl methylphosphonate (THMP) (0.13 mL) was added in another 13 mL of Milli-Q water and the pH was adjusted to 5.0–6.0. This solution was added to the particle dispersion and the mixture was stirred at 100 °C and 600 RPM under reflux overnight. Afterwards, particles were separated through centrifugation at 24,700× *g* for 10 min and washed twice with excess of ethanol and once with water. The PDSNPs were dried in oven at 80 °C and stored at room temperature for further use.

### 2.3. Synthesis of Amino-Propyl Modified DSNPs (ADSNPs)

The DSNPs were vacuum dried for 18 h before functionalization with 3-aminopropyl triethoxysilane (APTES) to remove any air pockets or moisture. Dried DSNPs (400 mg) were stirred at 500 RPM and the mixture was heated to 50 °C. The APTES (0.42 mL) was then added dropwise and the mixture was refluxed for 18 h. The ADSNPs were separated from the reaction mixture through centrifugation at 24,700× *g* for 10 min, washed twice with excess of acetone and once with water, and then dried overnight at 80 °C.

### 2.4. Synthesis of Succinic Anhydride Modified DSNPs (SDSNPs)

The ADSNPs were used for further modification with succinic anhydride (SA) [29]. The ADSNPs (100 mg) were dispersed in 40 mL of dimethylformamide (DMF) by sonication in water bath for 3 min. Succinic anhydride (SA, 900 mg) was dissolved in 10 mL of DMF and added to the ADNSPs’ dispersion and reaction was run overnight at 40 °C, 500 rpm. Then, acquired SDSNPs were centrifuged at 24,700× *g* for 10 min. The SDSNPs were washed using ethanol twice and water once and the particles were dried in an oven overnight.

### 2.5. Physicochemical Characterization of DSNPs and Derivatives

Particle size characterization was performed by using a Zetasizer Nano ZS at a back-scattering angle of 173° using disposable cuvettes. Particles at a concentration of 100 µg/mL were dispersed by sonication in a water bath for 5–10 min in water, pH 1.2, and 5.0 and 6.8 buffer as required in the study. A standard operating procedure was adopted based on the refractive index of the material (RI = 1.4) and dispersant. Every sample was run 100 times for 10 s each run in three sets. ζ-potential measurements were done using ζ-cell (1070, Malvern Panalytical, Malvern, UK). The DSNPs and derivatives were analyzed for organic content using thermogravimetric analysis (TGA). Each sample was analyzed within a temperature range from 25–900 °C at 10 °C/min in a 70 µL alumina (aluminum oxide) crucible. A blank crucible was used as a control. Solid-state NMR was performed on a Bruker Avance III 300 MHz spectrometer. The ^13^C cross polarized magic angle spinning (CPMAS), ^29^Si CPMAS, and ^29^Si single-pulse magic angle spinning (SPMAS) solid-state NMR (SSNMR) spectra were obtained using a 4 mm rotor spinning at 5 kHz. All chemical shifts are referenced to tetramethylsilane (TMS). For SPMAS, the relaxation delay was 120 s. Transmission electron microscopy (TEM) images were obtained on a Hitachi 7700 microscope operated at 100 kV. Pore dimensions, volume, and specific surface were obtained using nitrogen absorption (N_2_-BET) at Tristar, Micromeritics-II, 3020. The pore size distribution was measured from the adsorption branch of the isotherm using the BJH model. Standard curve of exenatide in pH 5.0 MES (2-(*N*-morpholino)ethanesulfonic acid) solution was prepared before proceeding with loading experiments by serially diluting 1000 µg/mL to 7.8 µg/mL (2× dilution at each step). A blank was run using buffer only vial. A C18 column having a diameter of 4 mm, pore size of 110 Å, and particle size of 5 µm (Phenomenex Inc., Torrance, CA, USA) was used for high-performance liquid chromatography (HPLC). A solution of potassium dihydrogen orthophosphate (20 mM) was prepared and adjusted to pH 2.5 using 85% *w*/*w* orthophosphoric acid. This was designated as solvent A and HPLC gradient grade acetonitrile was used as solvent B. Each standard or test sample was run using gradient flow settings at a flow rate of 1.5 mL/min at 25 °C for 30 min using Nexera i (Shimadzu, Kyoto, Japan) equipped with photodiode array (PDA). Area under the curves were measured at 215 nm.

### 2.6. Loading of Exenatide into Pristine and Modified DSNPs

Exenatide acetate solution and particle (DSNPs, ADSNPs, PDSNPs, MCM-41, SDSNPs) dispersions (4 mg/mL) were prepared in pH 5.0 MES buffer. Equal volumes (0.5 mL) of the two were mixed together in triplicate and allowed to stir at 350 rpm and rt for 90 min. After 90 min, exenatide-loaded particles were separated via centrifugation at 20,000× *g* RT for 10 min using a benchtop centrifuge. Pellets were used straight away or stored at −20 °C until needed for experiments. Initial and final concentrations were analyzed to determine loaded content via HPLC method mentioned above using the following formula.

$$\%\;loading=\left(\frac{Initial\;amount\;of\;exenatide\;added-exenatide\;supernatant}{Total\;mass\;of\;loaded\;particles}\right)\;\times 100$$

### 2.7. Coating of PDSNPs with Chitosan

A weight ratio of 0.5:1 of chitosan to NPs was used for coating purposes. Particles (2 mg) were dispersed in 1 mg/mL chitosan solution (1 mL). The chitosan solution (1 mg/mL) was either added to the separated pellet or stirred for 30 min at rt, 350 rpm or in two different sets of experiment where the chitosan solution was added at the initial and 90th minute during loading. After this step, coated particles were separated at 20,000× *g* for 10 min at rt. The coated particles were washed with 0.1 M acetic acid solution. The amounts of exenatide leached during coating were measured by analyzing the supernatant from each washing step. All experiments were performed in triplicate. Coated particles are denoted as CPDSNPs.

### 2.8. In Vitro Release of Exenatide from PDSNPs

The PDSNPs and CPDSNPs with 400 µg equivalent exenatide were dispersed in 5 mL of the united states pharmacopoeia (USP) specified simulated gastric fluid test solution without enzymes (pH 1.2) and simulated intestinal fluid test solution without enzymes USP (pH 6.8) and stirred at 150 rpm and 37 °C. Aliquots (200 µL) were taken at 0.08, 0.5, 1, 2, 4, 6, 8, and 24 h and centrifuged at 20,000× *g* for 3 min. Equal volume of fresh buffers (pH 1.2 and 6.8) were added to rinse the pellet and put back the suspension to the total volume of release buffer. Samples were analyzed using the established HPLC method explained in the previous section. All experiments were performed in triplicate.

### 2.9. In Vitro Permeability Assay of Exenatide- and Exenatide-Loaded PDSNPs Using a Caco-2 Monolayer Model

The Caco-2 cells (P-4) were seeded at a density of 1 × 10^5^ cells/well in 12 trans-well insert (0.4 μm pore diameter, 1.12 cm^2^ area) (Corning Inc., Kennebunk, ME, USA) plates and were grown in Minimal Essential Medium (MEM, 1X) supplemented with 10% fetal bovine serum (FBS) +1 *v*/*v*% glutamine +1 *v*/*v*% pen–strep until the transepithelial electrical resistance (TEER) value reached 400–600 Ω cm^2^. TEER measurements were recorded using an electrode connected to an EVOM volt-ohmmeter (World Precision Instruments, Sarasota, FL, USA). At the time of experiment, using HBSS-HEPES buffer pH 6.5 and 40 µg/mL equivalent of exenatide solution, the PDSNPS and CPDNSPs in the buffer were added separately in triplicate in respective apical chambers. The HBSS-HEPES buffer pH 7.4 (1.5 mL) was added in the basolateral chamber. Samples (750 µL) were collected at 0.5, 1, and 2 h. After 2 h, treatment was removed. The TEER measurements were made at 0, 0.5, 1, 2, 6, and 24 h. Samples were analyzed using the Human Exendin-4 ELISA Kit (Creative Diagnostics, Upton, NY, USA; Cat No: DEIA-BJ815) following the supplier’s guidelines.

### 2.10. In Vitro Permeability Assay of Exenatide- and Exenatide-Loaded PDSNPs Using Caco-2/HT29-MTX/Raji-B Triple Co-Culture Model

The Caco-2 and Raji B were purchased from American Type Culture Collection (ATCC, Manassas, VA, USA). The HT29-MTX cell line was kindly provided by T. Lesuffleur (INSERM U178, Villejuif, France). The Caco-2 (passage number P-62), HT29-MTX (P-40), and Raji B cells (P-19) were grown separately in tissue culture flasks (Orange Scientific, Braine-l’Alleud, Belgium), in Dulbecco’s modified Eagle’s medium (DMEM) with ultraglutamine (Lonza, Vervieres, Belgium) supplemented with 10% (*v*/*v*) of inactivated fetal bovine serum (Biochrom GmbH, Berlin, Germany), 1% (*v*/*v*) of non-essential aminoacids 100× (Biochrom GmbH, Berlin, Germany), and 1% (*v*/*v*) of antibiotic mixture (final concentration of 100 U/mL penicillin and 100 U/mL of streptomycin) (BioWest, Biowest Europe, Nuaillé, France). Cells were sub-cultured once a week with 0.25% trypsin–EDTA (1×) and seeded at a density of 5 × 10^5^ cells per 75 cm^2^ flask. The culture medium was replaced every other day. Cells were maintained in an incubator at 37 °C and 5% CO_2_ in water saturated atmosphere. The epithelial permeability of free exenatide and loaded exenatide in PDSNPS and CPDSNPs was also assessed on a Caco-2/HT29-MTX/Raji-B triple co-culture model grown in DMEM, +10% FBS, +1% pen/strep, +1% non-essential amino acids [30]. The Caco-2 and HT29-MTX in a proportion of 90:10 were seeded at a final density of 1 × 10^5^ cells/well on 0.4 µm polyester membrane Transwell^®^ inserts with a cell growth area of 1.20 cm^2^ (Corning Inc., Kennebunk, ME, USA) and allowed to grow for 21 days with medium changes every other day. At day 14, 1 × 10^5^ Raji B cells were added to the basolateral compartment of the Caco-2/HT29-MTX model in order to promote the differentiation of Caco-2 cells into M cells. On the day of the experiments, the medium was removed from the apical and basolateral compartments and the cells were washed twice with the HBSS pre-heated at 37 °C. Fresh HBSS (pH 7.4) was added to both compartments and the system was allowed to equilibrate for 30 min at 37 °C under agitation using an orbital shaker (100 rpm). After that, the HBSS from the apical compartment was replaced by 0.5 mL of exenatide solution, PDSNPs, and CPDSNPs dispersed in pre-warmed HBSS (pH 6.5) at a concentration of 40 µg/mL of exenatide. Sampling intervals and analysis of samples were the same as used in the Caco-2 monolayer system permeability assay.

### 2.11. Statistical Analysis

All experiments were done in triplicate unless otherwise stated. Datasets were analyzed using one-way ANOVA and post-hoc Tukey’s test where applicable.

## 3. Results and Discussion

### 3.1. Preparation and Characterization of Pristine and Modified DSNPs

**Figure 1:**
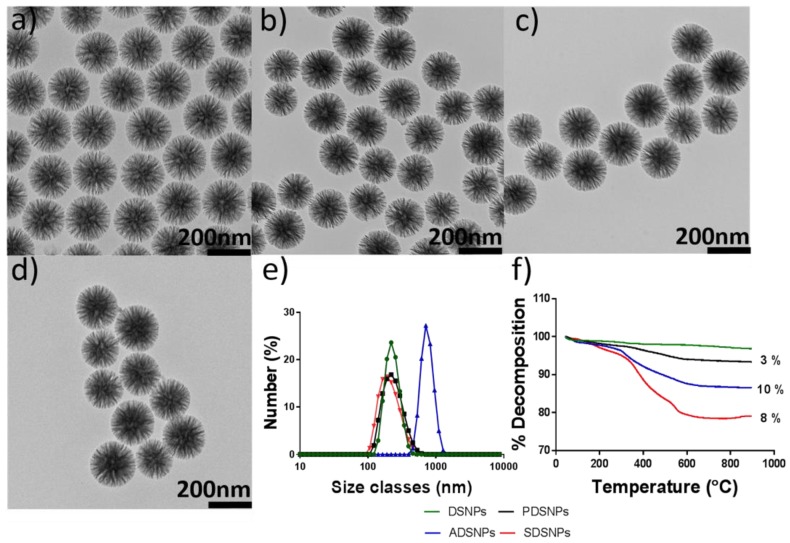
(**a**–**d**) TEM images of pristine DSNPs, PDSNPs, ADSNPs, and SDSNPs, respectively, showing dendritic morphology with pores radiating from the center; (**e**) particle size distribution showing that ADSNPs follow the trend of aggregation and remaining negatively charged DSNPs exhibit particle size at pH 7.4, agreeable with that of TEM imaging; (**f**) TGA of pristine and functionalized DSNPs.

The DSNPs were prepared using a bi-continuous microemulsion approach in which a ratio of oil–water of 1 was maintained [31]. The TEOS-containing organic solvent phase, chlorobenzene formed an emulsion with the lower aqueous phase. The surfactant CTAC and base TEA acted as pore template and catalyst, respectively, and chlorobenzene was used as a pore expanding agent [32]. Based on the synthesis approach for preparation of DSNPs, a typical morphology with wrinkle-like radial pores was obtained. Final morphology of dendritic-like pore structures was studied via TEM (Figure 1a). Analysis of TEM images shows uniform spherical particles of around 180 nm with radial pores originating from the center. The size measurement from TEM was slightly lower than that from DLS (226 nm) for DSNPs, as the latter measures hydrodynamic diameter which is the size of a hypothetical hard sphere diffusing in the same manner as the particles, being measured in a suspension [33]. A stronger negative surface charge of the particles may be helpful to improve colloidal stability in physiological conditions [34]. For this purpose, succinyl (–CO(CH_2_)_2_COO^−^) and methyl phosphonate (–CH_2_-PO^−^_3_) modifications were chosen where electronegative carboxyl groups and phosphonate groups can impart stronger electronegativity relative to that imparted by silanol groups under the studied pH conditions [35]. Amino-propyl-group-modified DSNPs were prepared to provide the chemical basis for succinic modification and to see the possible effects of surface-charge variation on loading of exenatide as amino-propyl DSNPs have a positive surface charge in contrast to DSNPs, PDSNPs, and SDSNPs. Consequently, a library of modified DSNPs was prepared to screen the functionalized DSNPs for suitability to use as candidate for oral exenatide delivery systems. Details of particle size, ζ-potential, and polydispersity index (PDI) are summarized in Table 1. All particles had a hydrodynamic diameter range of ~250 nm except amino functionalized particles which showed a largest mean size of ~740 nm. Particles were more homogenously dispersed due to the fact of their enhanced surface charge using succinyl and methyl phosphonate groups shown by high ζ-potential −23.2 mV and −30.5 mV and PDI 0.15 and 0.26, respectively, as compared to silanol containing DSNPs with relatively weaker ζ-potential of −17.1 mV and PDI 0.34 at pH 7.4. It was interesting to note that at the same pH ADSNPs were aggregated to size around 0.7 µm corresponding to relatively less magnitude of ζ-potential (11 mV) and, hence, low repulsion observed for positively charged particles at the given pH [36]. The thermograms from TGA provided insight into the extent of organic modification on DSNPs. The TGA analysis showed that phosphonate-, amino propyl-, and succinyl-functional groups made up a total of 3%, 10%, and 8% organic content, respectively (Figure 1e) confirming successful modification. Here, it is important to mention that calculations were made by subtracting the % organic content of preceding functionalization, i.e., succinyl group modification was shown to have 21% organic content, while amino propyl modification had 13% organic content, so the difference of these two values was 8% giving value to succinyl modification.

The presence of specific functional groups on DSNP surfaces were confirmed using ^13^C CPMAS, ^29^Si CPMAS, and ^29^Si SP MAS spectra. Spectra of pristine DSNPs from ^29^Si SP solid-state NMR confirmed resonance peaks for Q_4_ (δ = −120 ppm) and Q_3_ (δ = −102 ppm) structures (Figure S1a) [31,37]. The Q3 and Q2 structures represent silicon sites (atom) bound via siloxane bridges to another three or two Si atoms, respectively [38]. Such structures terminate the three-dimensional structure of silica and their high levels are necessary for electrostatic adsorption of drug molecules and further functionalization. The (Q2 + Q3)/Q4 ratio provides fractions of silanols (0.21 for DSNPs) available for electrostatic binding [34,39]. The ^13^C CP/MAS and FTIR spectra for all modifications were recorded (Figures S1 and S2). In the case of ADNSPs, the NMR peaks at 9.0, 21.2, and 42.5 ppm were observed which corresponded to the amino-propyl group bound to DSNPs (Figure S1b). Furthermore, small peaks at 29 and 58 ppm show diminished amounts of ethoxy groups which confirmed that the particles were functionalized through ethoxy groups and letting aminopropyl groups free for further modification [40]. This was confirmed by FTIR, where N–H stretching of primary amine at 3516 cm^−1^ could be observed (Figure S2). Succinic groups were appended on ADSNPs using a ring opening linker elongation reaction of the SA with amine group [41]. Peak at 178 ppm confirmed attachment of carbonyl carbon, within the carboxyl group, to the amino-propyl group of ADSNPs. An O–H bending of carboxylic acid appended to ADSNPs was also confirmed at 1390 cm^−1^ via FTIR (Figure S2). Phosphonate functionalization (PDSNPs) was directly performed on DSNPs. Grafting of phosphonate silane on DSNPs was noted by identifying THMP peaks at 10, 21, and 60 ppm on SSNMR spectra corresponding to the structures shown in Figure 1b [34].

As particles were functionalized with groups of various spatial conformations, it was important to study their porosity and textural properties, knowing that functionalization may render limited space to load exenatide. Surface area, pore size, and pore volume were studied using nitrogen sorption analysis. It was found that surface area decreased with every increment of functional group upon pristine DSNP (Table 1). For the average monolayer density, 2.1–4.0 amino propyl groups are found per square nanometer of modified surface which implies that modification will lead to occupation of pores and reduction of surface area to an extent relative to the number of functional groups appended [42]. Amino-propyl groups were subsequently functionalized by carboxyl groups, which led to further reduction in surface area (83 m^2^/g), as well as pore size and lower loading of exenatide shown later in the loading results. Similarly, functionalization with propyl methylphosphonate silane has been reported to reduce the surface area as is seen in the current study [43]. However, PDSNPs still had the highest available surface area among functionalized DSNPs (305 m^2^/g). Pore sizes read from each sample indicated a pore size of ~11 nm in the case of DSNPs, PDSNPs, and ADSNPs.

Likewise, functionalization of different groups significantly decreased the pore volume from 1.14 cm^3^/g for DSNPs followed by PDSNPs (0.88 cm^3^/g), ADSNPs (0.60 cm^3^/g) and lowest with SDSNPs (0.39 cm^3^/g). It can be postulated that high loading can take place provided enough pore size entrance is present to capitulate pore volumes. The size of exenatide (molecular weight related diameter calculated from empirical results of several model proteins and enzymes) is 2.3 nm and pore entrance (i.e., pore as an opening/ bottle neck for broader channel downwards as in the case of DSNPs) is much larger (11 nm) compared to the theoretical diameter of exenatide. It is also important to note that a conventional MSN, MCM-41 having merely a pore size of 2–3 nm, may not be a suitable candidate for the loading of peptide, which was also tested in our study [44].

### 3.2. Loading of Exenatide into Pristine and Modified DSNPs

**Figure 2:**
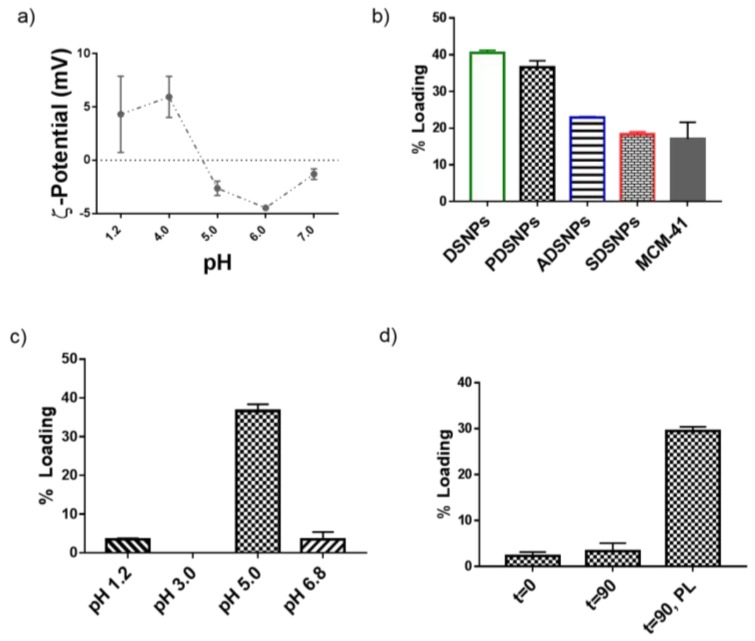
(**a**) ζ-potential profile of exenatide acetate at different pH confirming p*I*. (**b**) Loading of exenatide on different DSNPs and MCM-41, using pH 5.0 MES buffer, observed as maximum for pristine DSNPs. (**c**) Loading of exenatide on PDSNPs at different pH. (**d**) Effect of coating, chitosan in 0.1 M acetic acid solution (pH 3.8), strategies on exenatide loading. *t* = 0 and *t* = 90 shows the time interval at which chitosan was introduced into the exenatide loading solution. *t* = 90, PL (post loading) stands for the technique in which loaded particles were separated before the coating step. (All data are *n* = 3, Mean ± SD).

Exenatide is a 39 amino acid peptide with an isoelectric point (p*I*) of 4.86, as confirmed in Figure 2a [45]. It has been reported that the maximum loading of peptides onto SNPs generally takes place at their isoelectric point [46,47]. This is attributed to their neutral character and potential aggregation within pores of silica nanoparticles to achieve high loading at its p*I*. The loading was performed on all types of particles prepared in the study, as a step of screening the optimum candidate to test for potential application in oral delivery of exenatide. It was clear from the results that loading was related to available pore size and volume (Figure 2b, Table 1). The DSNPs, PDSNPs, ADSNPs, and SDSNPs demonstrated loading of 40%, 35%, 25%, and 18% by weight, respectively. For SDSNPs, LC was comparable to that of MCM-41 (<20%) indicating a correlation with the pore size that was not as big as it was in other modified DSNPs. At this point, PDSNPs were chosen based on the high charge surface required for better colloidal profile and high % loading of exenatide post functionalization.

The ζ-potential versus pH profile of exenatide was studied and it was found that charge on the peptide molecules was in a narrow range from −5.0 to 5.0 mV from pH 1.2 to pH 7.0 with confirmation of p*I* at around 4.9 (Figure 2a). This experiment was particularly helpful to establish an understanding of affinity of exenatide molecules at a defined pH range. Results from the loading experiment indicated that higher (~35%) loading could only take place at pH 5.0. Comparatively, less than 5% loading was seen at pH 1.2 and 6.8 and no loading could be observed at pH 3.0. This confirmed the hypothesis that effective loading could take place near iso-electric points that the pH of loading solutions had an effect on the binding affinity of exenatide molecules with PDSNPs.

The literature suggests that stronger electronegativity of oxygen within a phosphonate functional group contributes to stronger interaction with cationic counterparts [35]. Chitosan is a cationic that is naturally biodegradable, approved for medical use by the FDA, and a carbohydrate polymer that has been used for oral delivery of biologics [48]. Its cationic nature has been successfully exploited in preparing orally deliverable nano-complexes driven by strong electrostatic interactions [49,50]. Chitosan was coated on PDSNPs, because, due to the fact of their open pore structure, they have a tendency to leak prematurely and, if not properly controlled, this could lead to significant amounts of payload loss rendering limited application of large pore PDSNPs. It was hypothesized that due to the fact of strong positive surface charge and high molecular weight, chitosan molecules would be able to guard the adsorbed exenatide molecules on negatively charged PDSNPs. Three different methods were assessed for coating of chitosan on exenatide loaded particles. In one approach, loaded PDSNPs (post loading) were separated from loading solution and were stirred in chitosan solution for 20 min. In other two approaches, chitosan solution was introduced into loading solution either at the start of exenatide adsorption (simultaneous loading, *t* = 0 or *t* = 90 of loading for another 20 min. However, in the latter two methods where chitosan was co-introduced with exenatide solution, loaded particles contained only 2–4% of exenatide (Figure 2d). As chitosan is strongly positively charged at pH 3.8 (ζ-potential: +25 mV), it was expected that it would have strong electrostatic interaction with phosphonate groups on PDSNPs and could compete with exenatide at the same pH which could be the reason for low loading. Moreover, aggregation of exenatide at pH near its p*I* could also be reduced due to presence of the chitosan used in the study. Overall, loading was the highest with the post-loading coating process as the highest payload was found to be protected by coating with chitosan in this approach (Figure 2d). So, the post-loading method was adopted for further studies to prepare CPDNSPs.

### 3.3. Particle Size and ζ-Potential Analysis of PDSNPs and CPDNSPs at Different pH

**Figure 3:**
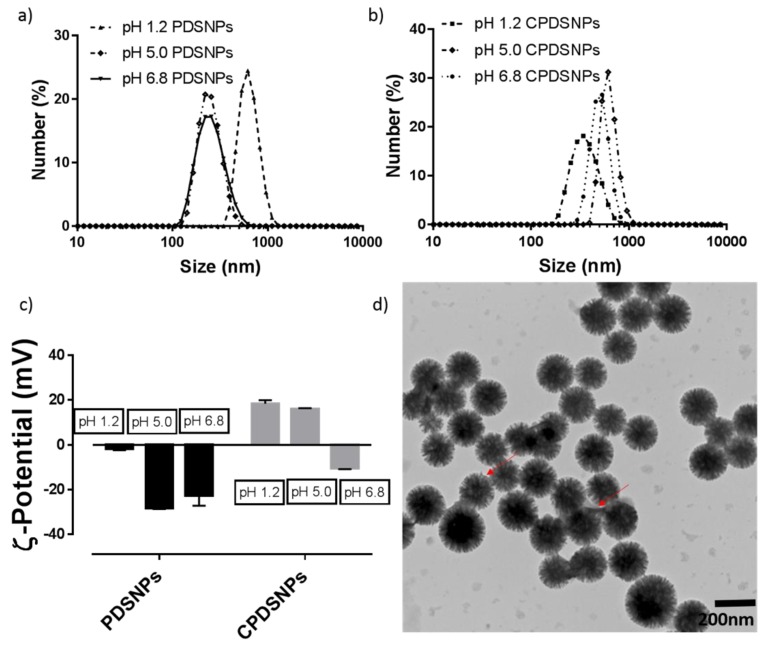
(**a**,**b**) The hydrodynamic sizes of uncoated and chitosan-coated PDSNPs behaved oppositely, indicating different assembly of CDSNPs, as the latter tended to aggregate as pH increased. (**c**) ζ-potential profile of the particles. (All data are *n* = 3, mean ± SD). (**d**) TEM image of CPDSNPs; radial pore network was clearly masked upon coating with chitosan.

In order to confirm attachment of chitosan onto PDSNPs, particles size and ζ-potential of both coated and uncoated particles was measured as a function of pH. It can be seen in Figure 3 that with an increase in pH from 1.2 to 6.8, the hydrodynamic diameter of PDSNPs tended to decrease, indicating aggregation of PDSNPs at pH 1.2 (615 nm, PDI: 0.46) and good dispersion at pH 6.8 (~250 nm). If ζ-potential is considered, this also correlates with the trend in aggregation behavior at lower pH, as particles carry less charge (−1.2 mV) at pH 1.2, leading to least repulsion and, hence, aggregation. A decrease in ζ-potential to −25 mV at pH 5.0 and 6.8 showed that they remained well dispersed due to the fact of repulsion among PDSNPs with higher pKa in the phosphonate group [51].

Chitosan-coated PDSNPs demonstrated a higher hydrodynamic diameter of ~740 nm (PDI: 0.5) at pH 6.9, while at pH 1.2 (PDI: 0.17) they appeared to be relatively dispersed with a size of 340 nm. A larger hydrodynamic diameter could be associated with amine groups which are less protonated at pH 6.8, leading to weak electrostatic interactions and aggregation [52]. This finding also coincides with ζ-potential findings for medium–high molecular weight chitosan of −11 mV at pH 7.0 [53]. A lower ζ-potential value also correlates with exposure of negatively charged particles due to the weakening electrostatic interactions at pH 6.8. Coating of chitosan was evident by reversal in ζ-potential of PDSNPS from −25 to +20 mV (pH 1.2 and 5.0), via TEM and TGA analysis (Figure 3, Figure S3).

### 3.4. In Vitro Release Study of Exenatide from PDSNPs and CPDSNPs

**Figure 4:**
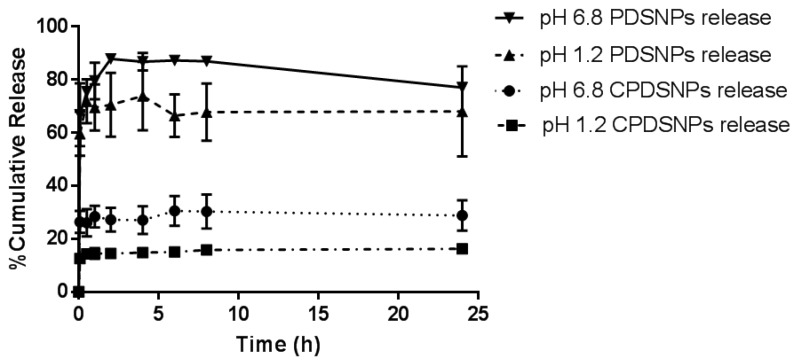
In vitro release profile of exenatide from PDSNPs and CPDSNPs at pH 1.2 and 6.8 for 6 and 8 h, respectively (all data are *n* = 3, mean ± SD).

As seen from Figure 4, PDSNPs released more exenatide in pH 6.8 buffer compared to pH 1.2, with similar level of burst release in the first 30 min irrespective of the pH of the solution. Over 70% of loaded content was released within an hour at simulated gastric pH and around 80% was found in pH 6.8 over a period of 2 h from PDSNPs. In the case of CPDSNPs, a sustained release pattern was indicated where only 18% of release was maintained over 24 h in pH 1.2 buffer. It was interesting to note that the release trend remained similar when it came to release from coated or uncoated particles at different pH. Unexpectedly, chitosan-coated particles also showed an initial burst with relatively less exenatide release in the first 30 min. The pattern of overall release showed incomplete release, which is reported to occur as equilibrium may be achieved between peptide in solution and the content in porous SNPs by displacement of exenatide molecules with solvent molecules flowing within channels of PDSNPs. The desorbed peptide molecules supposedly enter the solution and super-saturation is created [54]. Additionally, there is a significant amount which binds irreversibly to the surface of the particles and believed as a consequence of the hydrophobic interactions between hydrophobic amino acids and methylphosphonate on particles, which may lead to an incomplete release profile [55].

As seen from ζ-potential profiles in the previous section, it is clear that chitosan-coated PDSNPs have also a negative surface charge (~−11 mV) at pH 6.8. This finding is in line with previous reports that NPs with electrostatic coating tend to show the surface charge of core materials as PDSNPs did in the current study at a particular pH [56]. The release profile from electrostatically adsorbed chitosan show decreased burst release; however, only ~30% of the total drug was released from coated particles at pH 6.8 in 24 h. This remains a challenge in our study and further investigation into the role of chitosan physically adsorbed on PDSNPs to reduce the burst release is required.

### 3.5. In Vitro Permeation of Exenatide-Loaded PDSNPs and Coated PDSNPs

Intestinal permeability of hydrophilic macromolecules across intestinal epithelium is hampered by cellular tight junctions and lower residence time in addition to degradation in lumen. Exenatide is known as a poorly permeable peptide and multiple efforts are in progress to improve its permeation through intestinal barrier [9,57]. In the current study, permeation of exenatide solution was compared with that of exenatide loaded in PDSNPs and CPDNSPs using Caco-2 monolayer cell culture model which has absorptive and secretory characteristics of intestinal epithelial cells [58].

**Figure 5:**
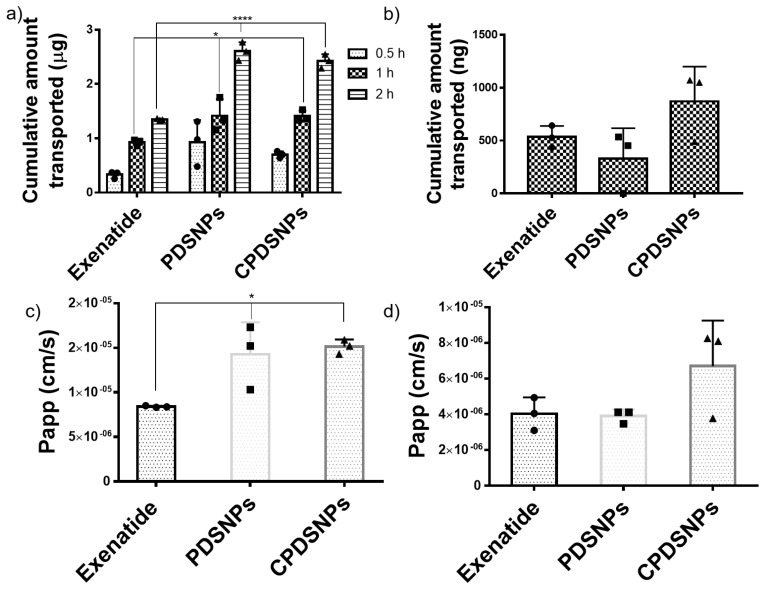
(**a**) Cumulative amount of exenatide transported through the Caco-2 monolayer at 0.5, 1, and 2 h, where monolayers were incubated with 40 µg/mL exenatide solution and equivalent exenatide-loaded nanoparticles (all data are *n* = 3, mean ± SD, and analyzed by one-way ANOVA, post-hoc Tukey’s test, * *p* < 0.05, **** *p* < 0.0001) (**b**) Cumulative amount of exenatide transported through the triple co-culture model at 2 h, where the cellular barrier was incubated with 40 µg/mL exenatide solution and equivalent exenatide-loaded nanoparticles. (**c**) Apparent permeability coefficient (Papp) of exenatide from PDSNPs and CPDSNPs at 2 h in the Caco-2 monolayer (all data are *n* = 3, mean ± SD, and analyzed by one-way ANOVA, post-hoc Tukey’s test, * *p* < 0.05). (**d**) Apparent permeability coefficient (Papp) of exenatide from PDSNPs and CPDSNPs at 2 h in (**d**) the triple co-culture model (all data are *n* = 3, mean ± SD).

Our assay in which Caco-2 monolayer was developed within 4–5 days was comparable in terms of duration with recent reports of a quick 3 day monolayer culture system used to study oral delivery of insulin and exenatide [10,57]. Data from Figure 5a indicate that compared to exenatide solution, PDSNPs and CPDSNPs significantly improved exenatide permeation at 1 and 2 h time points which validates our hypothesis of using the NPs to enhance transport with a 1.7 fold increase. The highest amount of exenatide transported at 2 h could be due to the transient opening of the tight junctions as seen from the decrease in TEER (Figure 5b). Interestingly, we did not see any significant difference in permeability of exenatide by coating PDSNPs with chitosan, reported to enhance the permeability of peptides enclosed in nanoparticles [59]. The significantly increased permeation of exenatide with PDSNPs and CPDSNPs compared to exenatide solution at 1 h of experiment was observed but at this time point there was no appreciable decrease in TEER value indicating closure of tight junctions. (Figure S4). This could indicate permeation was partly enhanced via transcytosis in which particles with exenatide could be endocytosed as vesicles with a size of more than 150 nm [60]. However, further studies with more complex models are required in order to elucidate the exact mechanism of transport using silica.

Findings from the Caco-2 monolayer model were complemented by testing free exenatide-loaded PDSNPs and CPDSNPs in the triple co-culture model (Figure 5b,d). The triple co-culture model was composed of Caco-2, mucus producing HT29-MTX and Raji B cells (lymphocytes), and closely mimicked the intestinal epithelial layer. This model can give epithelial permeability insights into peptides which have different cells contributing to physiological functions of the intestinal barrier including the presence of mucus [61]. In the triple co-culture model, at 2 h, CPDSNPs appeared to permeate relatively higher amounts of exenatide (Figure 5b) compared to both free exenatide and PDSNPs loaded with exenatide. In the triple co-culture model, chitosan-coated particles showed a 1.62 fold improvement as compared to free exenatide, which is in agreement with the 1.7 fold increase in the Caco-2 monolayer system. This observation could be correlated with the role of chitosan as a permeation enhancer, as chitosan is a mucoadhesive owing to the electrostatic interactions of the amine groups of the polymer with negatively charged glycoproteins within mucus [62]. It is also in agreement with a previous finding in which amine functional groups used to modify PEG-Biotin coating on nanoparticles led to a higher uptake of the functionalized NPs through a Caco-2/HT-29 co-culture model of intestinal permeability [63]. The triple co-culture model indicated the role of chitosan that could interact with mucins and enhance permeation of the CPDSNPs which was not the case in the Caco-2 monolayer model without mucus having relatively higher levels of exenatide transported. Additionally, the amounts of exenatide transported by PDSNPs across the Caco-2 monolayer were higher as compared to those found across the triple co-culture model. This could be attributed to the presence of mucin which added an additional barrier to decrease the transport physically and by repulsion due to the similar charge on mucins and PDSNPs [64].

## 4. Conclusions

This study reported successful synthesis of variously functionalized uniform large pore silica particles. The pH of the loading solution seemed to play an important role in dictating the affinity of peptide with PDSNPs confirming maximum exenatide loading achieved near its p*I* which was around pH 5. Among the tested particles, phosphonate functionalized particles showed 35% loading and were successfully coated with chitosan using electrostatic interactions with a post-loading method. Chitosan coating reduced the burst release of exenatide at both acidic and basic pH. In a Caco-2 in vitro model, we showed that both PDSNPs and CPDSNPs demonstrated significant improvement in permeability of exenatide compared to free exenatide. Furthermore, an in vitro triple co-culture model also revealed the role of chitosan coating where permeation of exenatide after 2 h was highest in CPDSNPs. Overall, this study provides a potential delivery system capable of improving permeability and in vitro relative bioavailability of biologics such as exenatide.

## Figures and Tables

**Table 1:** Hydrodynamic size, poly dispersity index (PDI), ζ-potential, and BET surface area of DSNPs and derivatives. (SD: standard deviation; BET: Brunauer–Emmett–Teller).

Dynamic Light Scattering			Nitrogen Sorption Analysis		
Particles	PDI (AU ± SD)	Particle Size (Mean Diameter nm ± SD)	ζ-Potential (mV)	BET Surface Area (m^2^/g)	Pore Volume (cm^3^/g)
DSNPs	0.34 ± 0.05	226 ± 9	−14.3	486	1.14
PDSNPs	0.26 ± 0.03	254 ± 7	−30.5	305	0.88
ADSNPs	0.45 ± 0.04	742 ± 13	11.5	191	0.60
SDSNPs	0.15 ± 0.01	248 ± 23	−23.2	83	0.39

## Supplementary Materials

The following are available online at https://www.mdpi.com/1999-4923/11/8/418/s1, Figure S1: (**a**) ^29^Si SP Solid State NMR spectra of pristine DSNPs. (**b**), (**c**) and (**d**) represent ^13^C CP/MAS Solid State NMR of PDSNPs, ADSNPs and SDSNPs respectively, Figure S2: FT-IR spectra of pristine and functionalised DSNPs, Figure S3. TGA evidence of chitosan coating on PDSNPs, Figure S4: Transepithelial electrical resistance (TEER) values of Caco-2 monolayer shown to recover after removal of treatment at 2 h. The recovery was monitored up to 24 h.
