# Supplementary material for: Rationally Designed Dendritic Silica Nanoparticles for Oral Delivery of Exenatide
Source: Pharmaceutics. 2019 Aug 19;11(8):418. doi: 10.3390/pharmaceutics11080418 (PMC6723263; doi:10.3390/pharmaceutics11080418)
Supplement: Supplementary file 1 [file pharmaceutics-11-00418-s001.pdf]

pharmaceutics

MDPI

Supplementary Materials: Rationally Designed Dendritic Silica Nanoparticles for Oral Delivery of Exenatide

Muhammad Mustafa Abeer, Anand Kumar Meka, Naisarg Pujara, Tushar Kumeria, Ekaterina Strounina, Rute Nunes, Ana Costa, Bruno Sarmento, Sumaira Z. Hasnain, Benjamin P. Ross and Amirali Popat

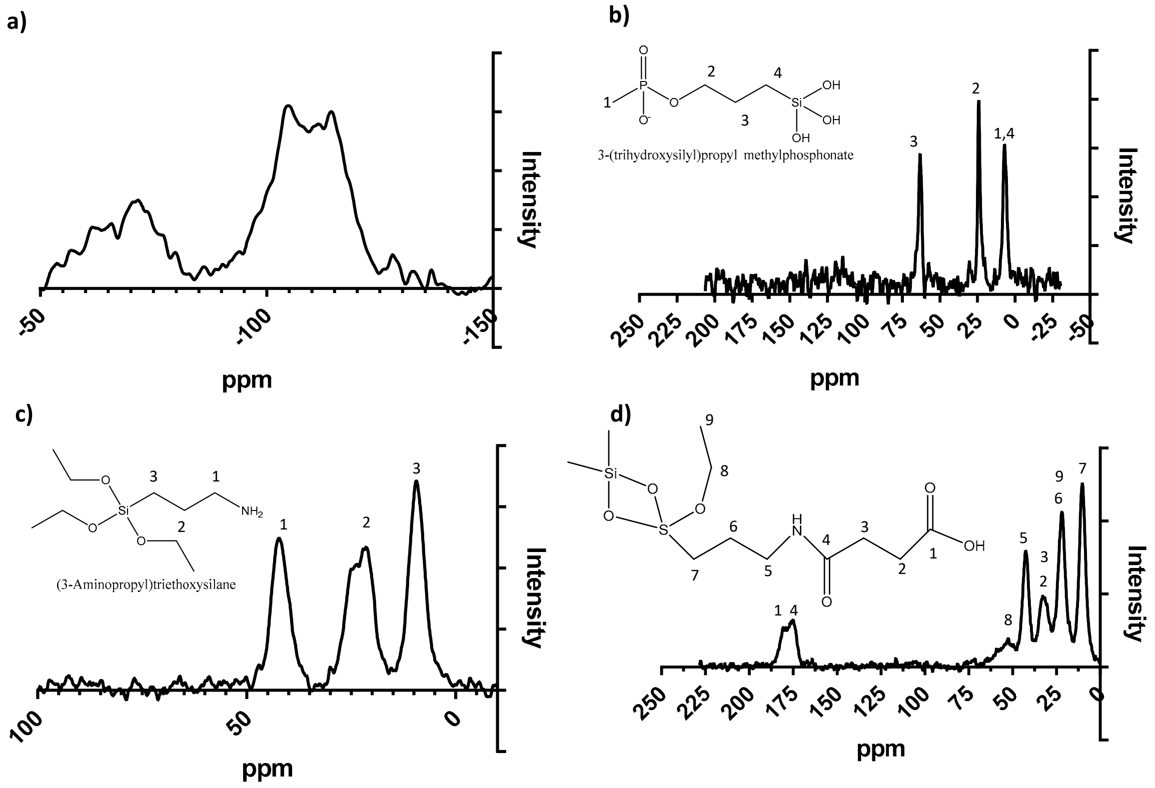

Figure S1. (**a**) ^{29}Si SP Solid State NMR spectra of pristine DSNPs. (**b**), (**c**) and (**d**) represent ^{13}C CP/MAS Solid State NMR of PDSNPs, ADSNPs and SDSNPs respectively.

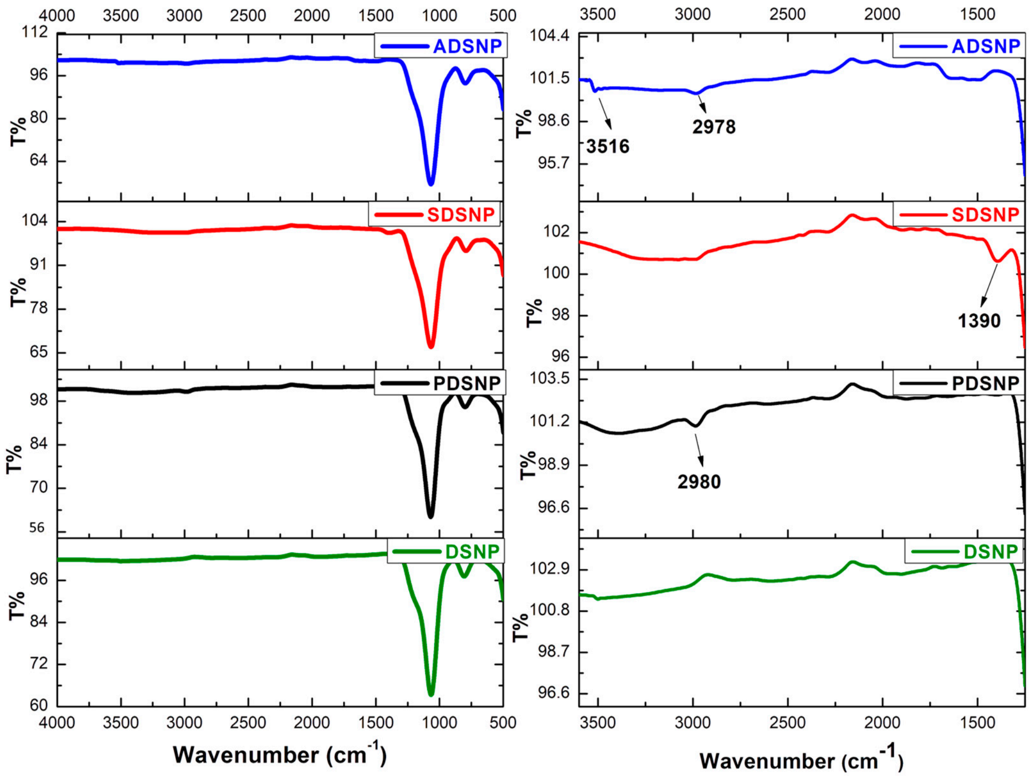

Figure S2. FT-IR spectra of pristine and functionalised DSNPs.

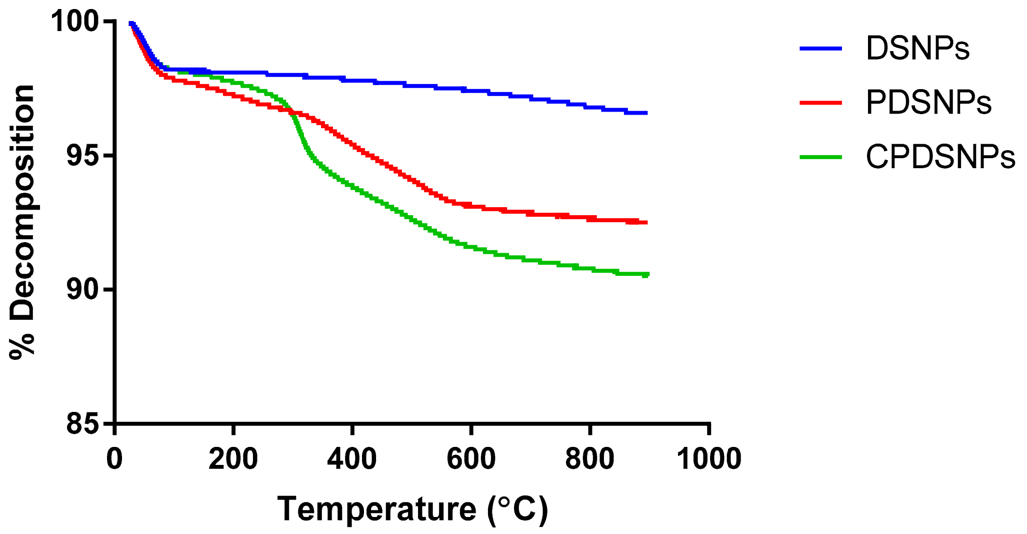

Figure S3. TGA evidence of chitosan coating on PDSNPs.

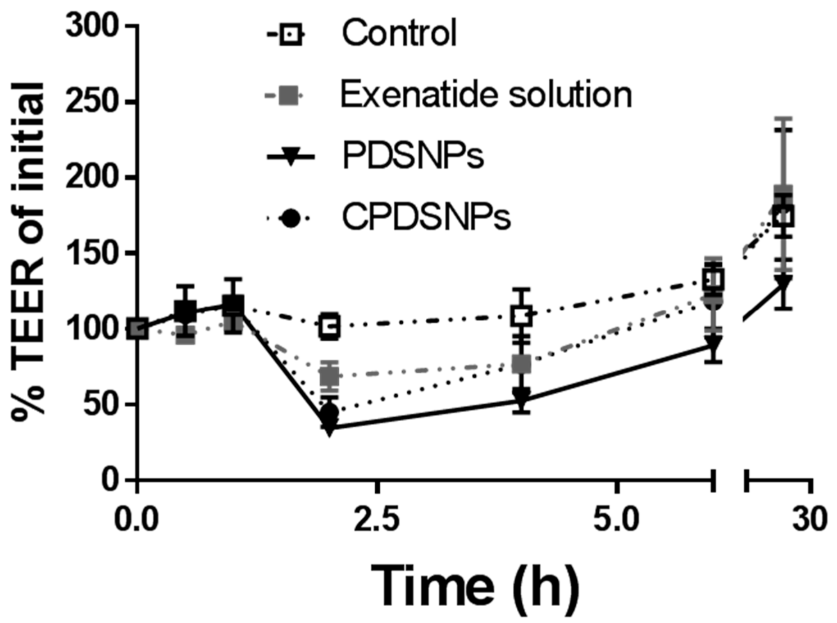

Figure S4. Transepithelial electrical resistance (TEER) values of Caco-2 monolayer shown to recover after removal of treatment at 2 h. The recovery was monitored up to 24 h.
